# Adaptive Knowledge Tracing with Dynamic Memory and Reinforcement Learning

**DOI:** 10.3390/s26061878

**Published:** 2026-03-17

**Authors:** Li Li, Zheng Duan, Zhi Zhou, Lian Liu

**Affiliations:** 1 College of Computer and Control Engineering, Northeast Forestry University, Harbin 150040, China; dzh@nefu.edu.cn (Z.D.); zhouzhi@nefu.edu.cn (Z.Z.); 2 School of Artificial Intelligence, Dalian Neusoft University of Information, Dalian 116023, China; liulian@neusoft.edu.cn

**Keywords:** knowledge tracing, forgetting behavior, reinforcement learning, Q-learning, dynamic memory network

## Abstract

Accurately assessing students’ knowledge states and dynamically adapting instructional interactions to their cognitive levels are fundamental to optimizing personalized learning. However, conventional knowledge tracing (KT) approaches are constrained by three critical limitations: data sparsity undermines prediction robustness, the neglect of forgetting behavior misrepresents real learning processes, and static knowledge-state modeling fails to capture learners’ dynamic cognitive changes. To overcome these shortcomings, this study proposes DRAKT (Dynamic Reinforcement learning-based Adaptive Knowledge Tracing), a novel model that introduces two key innovations: (1) a Q-learning-based knowledge-state adjustment mechanism, which dynamically updates mastery levels via a reward structure integrated with the Ebbinghaus forgetting curve; and (2) a dynamic memory update module that combines a gated recurrent unit (GRU) with attention-based filtering to capture long-term learning dependencies and suppress irrelevant memory traces. Experiments conducted on three public ASSISTments datasets (2009, 2012, and 2017) demonstrate that DRAKT consistently outperforms state-of-the-art baselines. On ASSISTments2017 and ASSISTments2009, DRAKT achieves AUC scores of 82.08% and 81.47%, respectively, surpassing the second-best model (GKT) by 2.75–6.57 percentage points in AUC and 4.77–5.75 percentage points in accuracy. In practice, DRAKT offers a reliable technical foundation for enabling personalized learning-path recommendation and real-time cognitive adaptation in intelligent educational systems.

## 1. Introduction

Currently, the field of education is undergoing a profound intelligent transformation. Adaptive learning systems and intelligent tutoring systems (ITS) have progressed from theoretical concepts to large-scale applications [1], deeply integrated into online learning platforms, blended classrooms, and even professional training scenarios. A core task of these systems is knowledge tracing, yet knowledge tracing models still face multiple challenges in practical applications [2]. Data sparsity constrains prediction accuracy, and most models treat knowledge points as independent units, neglecting the complex interrelationships among them. Traditional methods often assume static student knowledge states, whereas actual learning processes are influenced by various factors and continuously evolve dynamically, making it difficult for models to accurately reflect real learning conditions. Furthermore, due to the lack of dynamic adjustment mechanisms grounded in educational principles, existing models struggle to respond promptly to changes in learning states, limiting their effectiveness in personalized teaching scenarios.

Although researchers have proposed several improvement methods, the aforementioned issues continue to challenge the accuracy and applicability of model predictions, hindering the genuine realization of personalized learning.

To address the core limitations of existing knowledge tracing models, this study focuses on three explicit research questions (RQs):

RQ1: Can the Q-learning mechanism fused with the Ebbinghaus forgetting curve effectively address the static knowledge state issue and improve the dynamic tracking accuracy of students’ mastery levels? RQ2: How does the dynamic memory update module (integrating GRU and attention-based filtering) enhance the model’s robustness to data sparsity and ability to capture long-term learning dependencies? RQ3: Does the synergistic integration of reinforcement learning and dynamic memory modules enable DRAKT to generalize stably across datasets with different scales and learning behavior characteristics?

To address this, this paper proposes a Dynamic memory and Reinforcement learning-based Adaptive Knowledge Tracing model (DRAKT), aiming to resolve the problems of static knowledge representation and lack of dynamic adaptability in existing models. Specifically, DRAKT incorporates the Q-learning algorithm from reinforcement learning, achieving dynamic modeling and updating of students’ knowledge mastery states through a rationally designed reward mechanism. This mechanism provides corresponding positive or negative rewards based on students’ answering behaviors, thereby iteratively optimizing their knowledge mastery matrix. It not only clearly captures subtle changes during learning progress but also provides a reliable basis for personalized learning path planning. Additionally, this paper designs a dynamic memory update mechanism. By introducing an attention-weight-based memory read-write strategy, this module can capture long-term dependencies and reduce noise interference, significantly enhancing tracing accuracy and robustness. The DRAKT model combines dynamism and adaptability, making it particularly suitable for practical scenarios in online education where student states change frequently, thereby offering a novel solution for building efficient personalized teaching systems.

The main contributions of this paper are as follows:

- This paper introduces a Q-Learning reinforcement learning strategy, which dynamically adjusts the representation of knowledge points by updating the Q-values of knowledge states in real time and revises the degree of knowledge mastery based on the correctness of student responses. This enhances the model’s prediction accuracy during interaction.
- By designing a reward-guided memory update mechanism, the model can adjust memory content directionally in conjunction with reinforcement learning rewards. This module simulates the forgetting and review behaviors in real student learning, aligning more closely with the dynamic changes in knowledge states, thereby improving the reliability of state representation and the reasonableness of predictions.
- Experimental results show that DRAKT effectively enhances the model’s adaptive capability and prediction accuracy, outperforming traditional knowledge tracing models across three public datasets.

## 2. Related Work

Knowledge Tracing (KT) aims to automatically assess students’ knowledge states based on their historical learning data, thereby supporting personalized intelligent education [3].

Depending on the modeling approaches, current KT methods mainly fall into two categories: those based on traditional machine learning and those based on deep learning. Traditional KT models primarily include Bayesian Knowledge Tracing (BKT) and Item Response Theory (IRT) [4]. BKT [5] employs a Hidden Markov Model to infer students’ mastery of knowledge. While it offers strong theoretical interpretability, its simplicity limits its ability to capture complex learning characteristics. IRT accounts for item difficulty and individual student ability, aligning better with real educational contexts, but it often struggles to dynamically track knowledge state changes over time.

### 2.1. Deep Learning-Based Knowledge Tracing Models

With the rapid development of deep learning, models represented by Deep Knowledge Tracing (DKT) [6] have achieved significant breakthroughs. Piech et al. first utilized a Long Short-Term Memory (LSTM) network to construct the DKT model, effectively capturing the dynamic changes in students’ knowledge states and substantially improving prediction accuracy.

To further enhance model interpretability and the precision of estimating latent individual abilities, Yeung et al. [7] integrated IRT into the DKT framework, thereby improving its predictive capability. Subsequently, the Dynamic Key-Value Memory Network (DKVMN) [8], based on memory-augmented networks, emerged. By introducing a memory matrix that separately represents static knowledge concepts (Key) and dynamic knowledge states (Value), DKVMN enables more fine-grained knowledge state tracing. However, DKVMN is not fully effective in modeling long-term dependencies in sequential data. Building upon this, the SKVMN model [9] incorporates Hop-LSTM to capture dependencies among knowledge states at different timesteps within sequences, strengthening its ability to model long-term dependencies and significantly improving long-term knowledge state prediction performance. The Transformer model [10], proposed in 2017, has been widely applied across various domains due to its powerful parallel computing capability, self-attention mechanism, and exceptional ability to model long-range dependencies. As traditional DKT models have difficulty capturing long-term dependencies in sequences, Pu et al. [11] proposed the DKT + Transformer model, which uses the Transformer architecture to extract students’ hidden knowledge states, significantly improving computational efficiency while capturing long-term dependencies.

The performance of DKT models is highly susceptible to data quality. To address the generalization challenge posed by sparse student interaction sequences, Pandey et al. [12] introduced a self-attention mechanism to model students’ answer sequences, proposing the SAKT model to enhance generalization. Similarly, Ghosh et al. [13] proposed the Attention-based Knowledge Tracing (AKT) model, which uses an attention mechanism to weigh the influence of different historical responses on the current learning state. Other studies have attempted to improve prediction by enhancing the quality of exercise embeddings. The QIKT model [14], based on cognitive representations of questions, centers its student knowledge modeling around exercises, thereby enhancing model interpretability. Su et al. [15], addressing the lack of textual information, proposed a text-aware knowledge tracing model named EERNN. This model uses a bi-directional LSTM module to extract representations from exercise text and another LSTM module to combine this with representations of previously answered exercises to track students’ knowledge states. Liu et al. [16] further integrated exercise content with the learning process in their proposed EKT model, improving both prediction performance and interpretability. Tong et al. [17] argued that sequence-based models like DKT often overlook potential structural relationships among knowledge points. In their research, they incorporated a mechanism for influence propagation among knowledge points, enabling the model to consider dependencies and interactions between knowledge points when predicting student mastery states.

With the advancement of Graph Neural Networks (GNNs), some researchers have attempted to improve traditional KT models using graph structures. The introduction of Graph-based Knowledge Tracing (GKT) [18] provided a new approach to modeling the complex relationships between students and knowledge points, which traditional models found difficult. However, it still faces challenges such as data sparsity, training difficulties, and limited interpretability. To alleviate data sparsity, Yang et al. [19] introduced Graph Convolutional Networks (GCNs) to handle the mismatch between the number of knowledge points and exercises.

As educational data complexity increases, researchers have found that homogeneous graph structures are insufficient for fully expressing the multifaceted relationships among students, exercises, knowledge points, and courses. In recent years, heterogeneous graph methods have been introduced into KT tasks to more comprehensively capture the complex interactions between learning behaviors and knowledge structures. For instance, Jia Xu et al. [20] proposed an Heterogeneous Information Network (HIN)-based preprocessing method called HIN-KT, which provides a unified framework for modeling the multi-faceted interactions among students, exercises, and skills.

The evolution of KT models shows a trend from sequential modeling to multi-structural fusion. While early LSTM models could capture changes in knowledge states, they struggled with knowledge dependencies and complex semantics. The introduction of attention and memory mechanisms enhanced the modeling capability for long sequences and sparse data. Furthermore, GNNs and HINs have promoted the explicit multi-dimensional modeling of knowledge relationships, cognitive levels, and learning behaviors within models. This evolution not only improves prediction accuracy and interpretability but also lays a technical foundation for building personalized learning systems suited to real educational scenarios.

### 2.2. Forgetting-Aware Knowledge Tracing Models

On the other hand, the ubiquitous forgetting phenomenon in real learning processes has garnered increasing attention in recent years. Researchers have begun incorporating models of forgetting behavior to enhance the predictive performance and real-world applicability of KT models. Many deep learning-based KT models traditionally ignored the fact that students might forget previously learned content. To better capture this forgetting effect, Nagatani et al. [21] proposed the DKT + Forgetting model, which incorporates sequence-related forgetting features to enhance model performance, enabling a relatively accurate simulation of student learning and forgetting behaviors, thereby improving KT accuracy.

Addressing the issues that existing research often overlooks the dynamic nature of student knowledge states and the consistency of the learning process, Shuanghong Shen et al. [22] proposed improvements through the Learning Process-aware Knowledge Tracing (LPKT) model. LPKT employs mechanisms like learning gates and forgetting gates to directly model the student learning process, allowing for more precise monitoring of knowledge state changes and consequently improving prediction accuracy. Li Xiaoguang et al. [23] proposed the Learning and Forgetting-aware Knowledge Tracing (LFKT) model, which models forgetting behavior from perspectives such as time intervals and repetition counts, effectively enhancing the model’s ability to capture knowledge forgetting phenomena. Zheng Haodong et al. [24] noted that GKT does not consider student forgetting behavior or the temporal characteristics of answering the same knowledge point at different times, which affects prediction accuracy. They introduced cognitive assimilation theory to model the forgetting process in learning, enabling the model to predict learning outcomes over longer periods. Zhang Wei et al. [25] proposed the Cognitive Structure modeling and explicit Forgetting calculation for Knowledge Tracing (CSFKT) model. This model uses GNNs to dynamically update students’ cognitive structures and explicitly calculates the probability of knowledge forgetting, significantly improving the characterization of changes in student knowledge states. Zhang Zhaoli et al. [26], addressing the fact that students’ forgetting patterns are highly personalized rather than uniform, proposed the Personalized Knowledge Forgetting Tracing (PKFT) model, which incorporates student learning behavior features and ability features to model the forgetting process.

Addressing the shortcomings of existing models in neglecting knowledge point relationships and forgetting behavior modeling, Jia Rui et al. [27] proposed a deep knowledge tracing model that integrates knowledge point correlation and forgetting degree. They constructed a knowledge point correlation matrix using statistical methods and employed an attention mechanism to meticulously model the interaction of forgetting degrees, significantly improving predictive performance. Yan Qiuyan et al. [28], noting that existing models underutilize multi-modal information from questions and skills and ignore the impact of different modalities on memory levels, proposed the Multi-modal Forgetting-aware Knowledge Tracing (MFKT) model. MFKT combines multi-modal information to optimize the embeddings of question and skill nodes and integrates a forgetting mechanism to more accurately simulate student memory behavior. Addressing the lack of multi-dimensional, explicit difficulty modeling in existing models, MDKT [29] uses BERT + CNN to extract semantic difficulty, combines it with statistical and cognitive difficulty to form a multi-dimensional, explicit difficulty representation, and employs the multi-head attention mechanism of Transformer to achieve fine-grained prediction of student knowledge states.

In recent years, KT research has made significant progress in enhancing model prediction performance and real-world applicability. These efforts have collectively advanced the predictive accuracy and interpretability of KT models, providing a solid technical foundation for accurately identifying learners’ weak knowledge points and supporting personalized instructional interventions.

## 3. Materials and Methods

**Figure 1:**
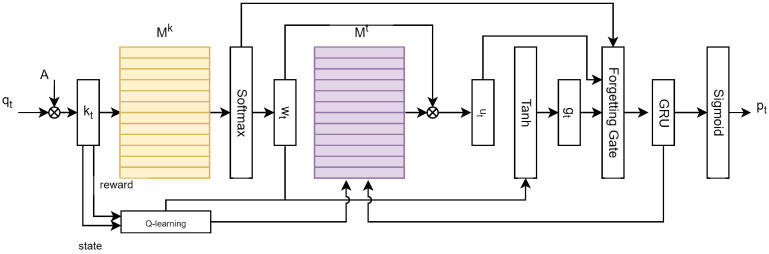
The Structure of DRAKT.

The DRAKT model is composed of five key modules: a knowledge input and representation module, a dynamic key-value memory network module, a reinforcement learning-based knowledge point adjustment module, a dynamic memory update mechanism, and a prediction module. The overall architecture of the model is illustrated in Figure 1.

### 3.1. Knowledge Input and Representation Module

First, to address the limitation of existing models which primarily rely on discrete question/answer labels and overlook the semantic associations between knowledge points, the model conducts a knowledge representation conversion on the input data. This module is designed to fuse semantic and behavioral features, thereby enhancing representation richness. It utilizes an embedding layer in deep learning to process the features of the questions answered by students, capturing not only the semantic connotation of individual knowledge points but also the potential connections between different knowledge points.

The question index $q_{t}\in \left\{1,2,\ldots ,m\right\}$ is mapped from a discrete identification format to a continuous knowledge representation space, so as to accurately and elaborately express the semantic features of each knowledge point (including the semantic connotation of the knowledge points themselves and the potential associations between knowledge points):

(1) $$q_{t}=E\left(q_{t}\right)$$

where *E* is a learnable embedding matrix, and $q_{t}$ refers to the question index at time step *t*.

The answer embedding layer operates similarly: the input sequence a∈{1,2,3,…,n} represents students’ answering behaviors, where $a_{t}\in \left\{0,1\right\}$ indicates whether the answer is correct. Subsequently, it is concatenated with the read feature and passed through a linear layer:

(2) $$e_{a}\left(t\right)=W_{a}\cdot \left[a_{t};f\left(t\right)\right]$$

where $f\left(t\right)\in R^{d}$ denotes the compressed feature after memory reading, and $W_{a}\in R^{d_{a}\times \left(1+d\right)}$ is a learnable embedding matrix.

By fusing the question semantic embedding and answer behavior embedding, Equations (1) and (2) comprehensively capture the features of students’ current answering behaviors in an all-round and multi-dimensional way. This embedding representation provides a semantic-rich and precisely-characterized underlying support for subsequent dynamic knowledge tracing, particularly in aspects such as accurately modeling the evolution of students’ knowledge states and efficiently identifying knowledge gaps.

### 3.2. Dynamic Key-Value Memory Network

This module employs a Dynamic Key-Value Memory Network to store the evolving states of student knowledge. Addressing a key limitation of prior models like DKVMN [8], which relies on fixed memory read-write rules and struggles to adapt to dynamic knowledge changes, our design decouples the storage of knowledge point features and student mastery levels. This separation enables fine-grained, adaptive state tracking. Conceptually, it treats a student’s knowledge system as a readable and writable memory bank. This bank not only stores the static features of the knowledge points themselves but also continuously updates a dynamic record of the student’s current mastery level for each point.

The key memory *K* is a matrix $M^{K}$ of dimension (N,d), where *N* represents the total number of knowledge points in the system, and *d* is the dimension of the embedding vector. The initial value of the key memory is a learnable matrix $M^{K}\in R^{N\times d}$, where each row $k_{i}$ is a static, learnable embedding vector that uniquely represents a specific knowledge point $c_{i}$.

The value memory *V* is a matrix $M^{V}$ of the same dimension (N,d), initialized as $M^{V}\in R^{N\times d}$. Each row $v_{i}$ in *V* no longer represents the knowledge point itself, but instead indicates the student’s mastery of the knowledge point $c_{i}$.

At each time step *t*, the model calculates the similarity between the current question embedding $q_{t}$ and all keys in the key memory *K*, generates an attention weight vector, and performs normalization to obtain $w_{t}$ (which represents the attention degree of each sample to the memory in the key memory):

(3) $$w_{t}=\mathrm{softmax}\left(q_{t}\cdot {\left(M^{K}\right)}^{T}\right)$$

Based on the attention weights, the content is read from the value memory matrix $M^{V}$ in a weighted manner, and summarized as the knowledge state at the current moment:

(4) $$u_{t}=\sum_{b}w_{t,b}\cdot M^{V}{\left(t\right)}_{b,m}$$

Here, $M^{V}{\left(t\right)}_{b,m}$ denotes the value feature of the *b*-th sample at the *m*-th memory position at time *t*, and $u_{t}$ is the read knowledge state (which will be used for subsequent feature fusion and prediction).

### 3.3. Knowledge Point Update Module Based on Reinforcement Learning

To address the limitation of traditional knowledge tracing methods, which passively record sequential data and lack a mechanism for active feedback adjustment, we adopt Q-learning—rather than alternatives such as SARSA or DQN—for dynamic knowledge state updating. As a model-free approach, Q-learning adapts naturally to the uncertainty inherent in learning processes without relying on pre-defined transition rules, making it particularly suitable for capturing diverse and evolving student response behaviors. Furthermore, moving beyond conventional binary reward schemes (e.g., +1 for a correct answer [21]), we integrate the Ebbinghaus forgetting curve into the reward design to quantify forgetting risk. This ensures that the reinforcement signals align with realistic learning-forgetting cycles and carry pedagogical meaning.

**Figure 2:**
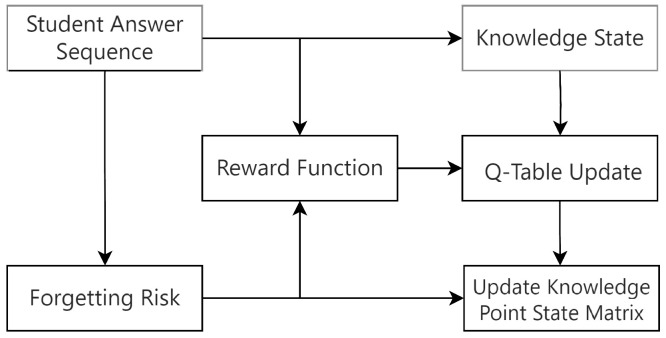
The Structure of knowledge update module based on Reinforcement Learning.

Specifically, Q-learning is used to estimate the value of state-action pairs (s,act), thereby simulating the essential “feedback–adjustment” loop observed in real learning. Guided by this education-aware reward mechanism, the model updates the corresponding knowledge representations in memory based on the dynamically computed values. As a result, the evolution of knowledge states more accurately and cognitively plausibly reflects how students internalize and consolidate knowledge over time. The structure of this reinforcement learning-based knowledge point update module is illustrated in Figure 2.

First, the state $s_{t}$ of reinforcement learning consists of the current question number $q_{t}$ and the answer $a_{t-1}$ to the previous question. The state is stored as a tuple and used as the index of the Q-table:

(5) $$s_{t}=\left(q_{t},a_{t-1}\right)$$

Actions correspond to the selection of memory positions in $M^{V}$, i.e., choosing which position to focus on updating:

(6) act∈A={0,1,2,…,ms}

where A denotes the action space, and ms is the number of memory positions in the dynamic memory network (used to update the mastery of knowledge points related to the current question).

Traditional deep knowledge tracing models usually frame learning sequence prediction as a typical supervised learning task, with the core optimization objective focusing on minimizing prediction errors. The traditional reward assignment method (simply setting the reward to 1 or −1 based on whether the answer is correct) can no longer meet the needs of personalized learning. Inspired by the Ebbinghaus forgetting curve, this module integrates the forgetting process into the reward function, dynamically adjusting the reward value according to the forgetting risk of knowledge points: the higher the forgetting risk, the greater the reward for successfully consolidating the knowledge point, and vice versa.

Inspired by the Ebbinghaus forgetting curve, which describes the exponential decay of memory over time, the reward is dynamically adjusted based on the quantified risk of knowledge forgetting. Specifically, for knowledge point *i* at time step *t*, its forgetting risk is defined by incorporating the Ebbinghaus forgetting curve formula as follows:

(7) $$R_{i}^{t}=\left(1-v_{i}^{t-1}\right)\cdot e^{-\mu \cdot \Delta t}$$

where $v_{i}^{t-1}$ is the mastery degree of knowledge point *i* at the previous time step, μ is the decay rate parameter of the Ebbinghaus forgetting curve, and Δt is the learning interval. The value of μ refers to the research of Zhang Nuan et al.: in current studies, this parameter is commonly set to 0.1 by default, so this study follows this value (μ=0.1). The value of the learning interval Δt is based on the research of Zhao Yajuan et al., determined by the average initial contact interval of similar students.

A supervised reward is designed, and the reward calculation is based on whether the current answer is correct: if the selected answer is correct, the reward is 1; if incorrect, the reward is −1. The specific definition formula is as follows:

(8) $$r_{t}^{1}=\left\{\begin{array}{cc} 1, & a_{t}=1\\ -1, & a_{t}=0 \end{array}\right.$$

This study redefines the reward function in reinforcement learning. This function combines the attention mechanism with the student learning forgetting risk model to form a comprehensive reward signal, driving the learning agent to consolidate memory more efficiently:

(9) $$r_{t}=r_{t}^{1}\cdot R_{i}^{t}$$

For each state-action pair, a vector of length $N_{q}$ (where $N_{q}$ is the total number of knowledge points) is stored in the Q-table. The model adjusts the Q-value according to the student’s answer result, and its iteration rule is defined as:

(10) $$Q\left(s_{t},act_{t}\right)=Q\left(s_{t},act_{t}\right)+\alpha \left[r_{t}+\gamma \max Q\left(s_{t+1},act^{\prime }\right)-Q\left(s_{t},act_{t}\right)\right]$$

where $r_{t}$ is the reward for answering the question, α is the learning rate (used to control the amplitude of each update), and γ is the discount factor (used to balance the immediate reward and future potential benefits). $\gamma \max Q(s^{\prime },act^{\prime })$ represents the maximum value of all optional actions under the next question state $s^{\prime }$. Through repeated iterations, the model can gradually learn the optimal decisions to take under different knowledge states, thereby dynamically adjusting the internal knowledge representation.

After the Q-learning module selects the action $act_{t}$, the corresponding Q-value $Q(s_{t},act_{t})$ is obtained, and it is converted into the attention gating coefficient for knowledge points through a nonlinear mapping:

(11) $$g_{t}=\sigma \left(\beta \cdot \tanh\left(Q(s_{t},act_{t})\right)\right)$$

where σ is the Sigmoid function, and β is the coefficient controlling the gating (used to adjust the attention weight of knowledge points). A higher Q-value means the knowledge point is more important in the current state, and the corresponding $g_{t}$ is larger. Then the gating coefficient is used to adjust the attention weight, and the specific formula is shown in Equation (12):

(12) $${\tilde{w}}_{t}=\mathrm{Softmax}\left(w_{t}+v_{t}\cdot \lambda g_{t}\right)$$

Here, $w_{t}$ is the attention weight, and λ is the coefficient that adjusts the influence intensity of the gating weight. The adjusted attention weight acts on the write vector $u_{t}$ to update the knowledge point mastery matrix $M^{V}$:

(13) $$M_{t+1}^{V}=\left(1-{\tilde{w}}_{t}\right)⊙M_{t}^{V}+{\tilde{w}}_{t}⊙u_{t}$$

This update rule shifts the global representation vector toward the Q-value direction under the current state, thereby strengthening the knowledge points with higher value in the current state and weakening those with lower value. This reinforcement learning-driven update strategy enables the model to no longer passively record students’ historical performance, but to actively correct the representation of knowledge states using feedback information, forming a mechanism similar to “strategic teaching”. In other words, while predicting students’ future answering performance, the model continuously improves its own cognition of students’ knowledge mastery, making the knowledge tracing process closer to the real learning and feedback cycle.

### 3.4. Dynamic Memory Update Mechanism

Memory update is achieved through two synergistic operations: dynamic memory updating and memory filtering. This integrated design addresses the issue of irrelevant memory accumulation over time, which would otherwise reduce model efficiency. Specifically, the filtering mechanism removes noisy information, while the dynamic update retains essential knowledge states and captures long-term dependencies. Together, these operations effectively simulate the selective retention and forgetting behaviors inherent in the learning process, ensuring the memory bank remains both efficient and pedagogically meaningful.

To enable the model to focus more on tasks highly relevant to the current knowledge points, a memory filtering mechanism is added to filter memories unrelated to the current task:

(14) $$V=\left\{v_{i}|\sigma \left(v_{i}\cdot q_{t}\right)>\theta \right\}$$

where θ denotes the filtering threshold.

Here, a forgetting gate and a reward signal mechanism are introduced to dynamically adjust students’ memory content. Bandura’s self-regulation theory points out that when people meet their self-set standards, they will use controllable rewards to strengthen and maintain their behaviors. Inspired by this, a reward matrix is introduced to adjust the forgetting gate strategy: when the reward signal is high, the forgetting gate will increase the weight of the current memory; otherwise, it will reduce the update weight. The adjustment of memory content is as follows:

(15) $$V=\left(1-\varepsilon \right)\cdot u_{t}+\varepsilon \cdot v_{t}+\eta \cdot r$$

where ε is the forgetting rate, and *r* is the reward matrix.

In addition, the Gated Recurrent Unit (GRU) is introduced into the memory update part to improve the model’s performance in task adaptability and long-term dependency handling. GRU is a variant of Recurrent Neural Network (RNN), designed to solve the gradient vanishing problem in traditional RNNs while better capturing long-term dependency relationships in sequences. Compared with Long Short-Term Memory (LSTM), GRU has fewer parameters and is more efficient in computation and storage. Introducing GRU in the memory update part enhances the model’s performance in task adaptability and long-term dependency handling; specifically, it is used to dynamically update the value memory *V* in the memory system, realizing accurate modeling of the memory update process by capturing the evolution of students’ knowledge states in different learning tasks.

**Figure 3:**
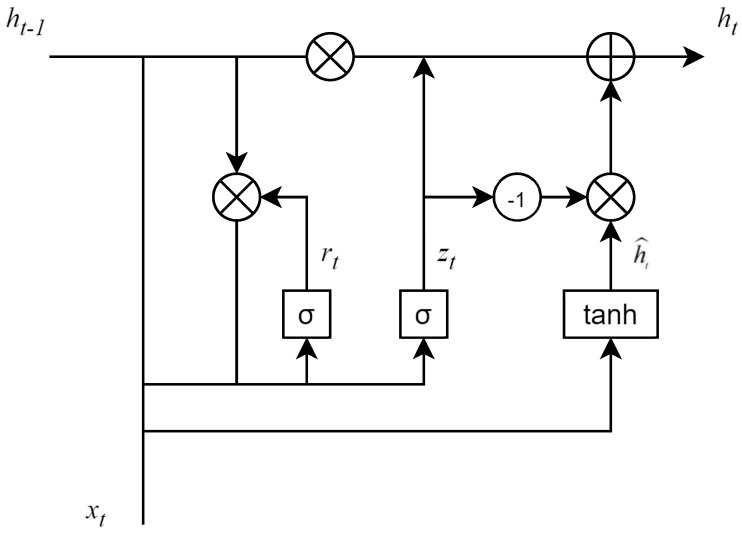
The Structure of GRU.

GRU consists of two components: an update gate and a reset gate, and its structure is shown in Figure 3. The update gate outputs a weight value in the interval [0, 1] via the sigmoid function: when the weight is close to 1, the model tends to retain the original memory information and integrate a small amount of new learning content; when the weight is close to 0, it discards the original memory information and focuses on absorbing knowledge from the new task. This dynamic balance mechanism allows GRU to accurately adapt to the memory update rules of students in different learning stages: it avoids model redundancy caused by excessive accumulation of old knowledge, while ensuring the effective integration of new information. The update process of GRU is shown in Equations (16)–(19):

(16) $$z_{t}=\sigma \left(W_{z}\left[h_{t-1},V\right]+b_{z}\right)$$

(17) $$r_{t}=\sigma \left(W_{r}\left[h_{t-1},V\right]+b_{r}\right)$$

(18) $${\tilde{h}}_{t}=\tanh\left(W_{h}\left[r_{t}\times h_{t-1},V\right]+b_{h}\right)$$

(19) $$h_{t}=z_{t}\times h_{t-1}+\left(1-z_{t}\right)\times {\tilde{h}}_{t}$$

Here, $z_{t}$ and $r_{t}$ denote the update gate and reset gate, respectively, and $h_{t-1}$ is the hidden state at the previous moment.

### 3.5. Prediction Module

The prediction module aims to accurately predict whether a student answers the current question correctly, based on the temporal evolution features of knowledge states. It maps the hidden state extracted by GRU to a prediction score Logits through a fully connected layer:

(20) $$\mathrm{Logits}\left(t\right)=W_{p}\cdot h_{t}+b_{p}$$

where $W_{p}$ is the weight matrix and $b_{p}$ is the bias term. Then, the Sigmoid function is used to convert it into an answer accuracy rate in the interval [0,1]:

(21) $${\hat{y}}_{t}=\sigma \left(\mathrm{Logits}(t)\right)$$

${\hat{y}}_{t}$ represents the predicted probability that the student answers correctly at time *t*; the closer the value is to 1, the higher the model’s confidence that “the student answers correctly”.

### 3.6. Model Training and Optimization

The model training process uses the standard binary cross-entropy loss function for optimization (the formula is shown below). The Adam optimizer is used for training; all weights of linear layers and embedding layers adopt the Kaiming normal initialization method, and biases are initialized to 0:

(22) $$W∼N\left(0,\frac{2}{d}\right)$$

This initialization method can adapt to network structures related to ReLU activation, effectively avoiding initial gradient vanishing or explosion, and accelerating model convergence. All bias terms are initialized to 0 to ensure no bias in the initial output.

The prediction task is to determine whether the student will answer correctly in the next attempt. During training, the model updates each parameter through the backpropagation algorithm, enabling it to gradually learn effective knowledge state representations and dynamic adjustment strategies:

(23) $$\ell =-\sum \left(y_{t}\log\left({\hat{y}}_{t}\right)+\left(1-y_{t}\right)\log\left(1-{\hat{y}}_{t}\right)\right)$$

## 4. Results

### 4.1. Dataset and Parameter Settings

The experiment leverages three public datasets from the online tutoring platform ASSISTment, namely ASSISTment2009, ASSISTment2012, and ASSISTment2017. Notably, the raw data of these datasets was collected from student interaction logs on the platform, capturing user answering behaviors and learning process records. In the data preprocessing stage, records with missing knowledge components, skill tags, or response outcomes were removed. Additionally, students with fewer than three answered questions were filtered out to ensure the completeness and validity of the data used for modeling. Specifically, the ASSISTment2009 dataset contains a substantial number of duplicate records; thus, invalid and redundant data have been filtered out by adopting the data processing method described in literature [30]. For the ASSISTment2012 and 2017 datasets, invalid data points were directly removed from the original sensor-collected data prior to experimental testing. Detailed specifications of the processed datasets are summarized in Table 1.

To ensure the reliability and stability of model evaluation and to mitigate overfitting, we adopt a ten-fold cross-validation approach. The specific procedure is as follows:

First, each dataset is randomly partitioned into 10 mutually exclusive subsets, ensuring a consistent distribution of students across all subsets to maintain data consistency. In each fold, 9 subsets are combined to form the training set. From this combined set, 80% of the data is used for model training, while the remaining 20% serves as a validation set for hyperparameter tuning (e.g., learning rate, memory filtering threshold). The remaining single subset is held out as the independent test set for the final performance evaluation. This process is repeated 10 times, with each subset used exactly once as the test set. Finally, the average performance across all 10 folds is reported as the final model performance metric.

The parameters of the model were configured through systematic grid search on the validation set to ensure reproducibility and optimal performance, with all experiments conducted under a unified hardware (NVIDIA RTX 2080Ti) and software (PyTorch 1.4.0) environment. The primary parameters of the model were set as follows: a learning rate of 0.0001, a batch size of 128, and training conducted over 50 epochs with early stopping triggered if validation loss did not decrease for 5 consecutive epochs. The question embedding dimension was set to 100 and the write vector dimension to 200, while the memory filtering threshold (θ) was fixed at 0.2. For the Q-learning component, a separate learning rate of 0.01 was applied along with a discount factor of 0.9 and an initial exploration rate of 0.2, which decayed exponentially during training to 0.05. These values were selected from defined search spaces—learning rate over [0.0001, 0.001, 0.01], batch size over [64, 128, 256], embedding dimensions over [50, 100, 200] and [100, 200, 300] respectively, epoch limits over [30, 50, 70], θ over [0.1, 0.2, 0.3, 0.4], Q-learning rate over [0.001, 0.01, 0.1], discount factor over [0.8, 0.9, 0.95], and exploration rate over [0.1, 0.2, 0.3] to balance model performance, training stability, and computational efficiency.

### 4.2. Comparison Algorithms and Evaluation Metrics

In knowledge tracing research, to comprehensively evaluate the performance of the proposed DRAKT model, several representative baseline models are selected for comparison, and the model performance is evaluated from multiple dimensions.

The characteristics of each baseline model are as follows:

- **BKT (Bayesian Knowledge Tracing)** [5]: As the most classic knowledge tracing model, it models learners’ knowledge states based on the Hidden Markov Model, and serves as the baseline for performance comparison in knowledge tracing models.
- **DKT (Deep Knowledge Tracing)** [6]: It first introduced Recurrent Neural Networks (RNNs) into the knowledge tracing task, which can better capture the temporal and semantic features in learning sequences, and its performance is significantly better than the traditional BKT.
- **AKT (Attention-based Knowledge Tracing)** [12]: It uses the attention mechanism to dynamically perceive students’ learning performance, enabling adaptive modeling of students’ different learning points, reflecting the application of the attention mechanism in knowledge tracing.
- **DKVMN (Dynamic Key-Value Memory Network)** [8]: It introduces the key-value memory network structure to explicitly store and dynamically update students’ mastery of different knowledge points, with significantly improved interpretability and flexibility compared to DKT and BKT.
- **DKT-Forget** [21]: It introduces the forgetting mechanism into DKT, enhancing the model’s adaptability to the dynamic changes of students’ knowledge states, and can more accurately reflect the knowledge points that students may forget; it is a knowledge tracing model based on dynamic forgetting mechanisms.
- **LPKT (Latent Performance Knowledge Tracing)** [22]: It jointly models the potential change process of knowledge mastery and its influencing factors through latent variables and pre-training mechanisms, further improving the prediction accuracy of knowledge tracing and verifying the performance of DRAKT under complex factors.
- **GKT (Graph-based Knowledge Tracing)** [18]: On the basis of DKT, it introduces the graph structure between knowledge points to explicitly model the complex relationships between knowledge points, which is suitable for large-scale educational data and can reflect the applicability of DRAKT on large-scale educational datasets.

To systematically evaluate the model performance, comparative experiments are conducted on three public datasets. The widely used evaluation indicators in knowledge tracing are adopted: AUC (Area Under the Curve) and ACC (Accuracy), which measure the classification performance to quantify the model’s performance.

### 4.3. Comparative Experiment Result Analysis

The experimental results (Table 2) on the three public datasets show that all evaluation metrics of the DRAKT model outperform those of the baseline models, with a performance improvement of over 2.75%. This result not only demonstrates DRAKT’s strong classification capability, but also verifies that its dynamic memory update module and memory filtering module can more effectively simulate students’ cognitive learning processes. This enables more accurate prediction of students’ future answer performance and enhances the overall performance of the knowledge tracing model.

To comprehensively evaluate the performance of DRAKT, we conducted comparative experiments against seven representative baseline models—BKT, DKT, AKT, DKVMN, DKT-Forget, LPKT, and GKT—on three public ASSISTment datasets (2009, 2012, and 2017). All models were carefully tuned via grid search on the validation set (details in Section 3.1) to ensure a fair comparison, using AUC (Area Under the Curve) and Accuracy (ACC) as evaluation metrics, with both reported to two decimal places.

As summarized in Table 2, DRAKT consistently outperforms all baseline models across every dataset. On ASSISTment2009, which contains small-scale and sparse data, DRAKT achieves an AUC of 0.81 and ACC of 0.80, surpassing the second-best model, GKT [17], by 2.75 and 4.77 percentage points, respectively. On the larger-scale ASSISTment2012 with massive interaction records, DRAKT attains an AUC of 0.81 and ACC of 0.80, exceeding GKT by 3.00 and 5.33 percentage points. For ASSISTment2017, which reflects more diverse learning behaviors, DRAKT reaches the highest scores with an AUC of 0.82 and ACC of 0.76, outperforming GKT by 6.57 and 5.75 percentage points.

Notably, DRAKT demonstrates stronger robustness compared to the baseline models. The fluctuation in its AUC across the three datasets is only 0.01, whereas models such as DKT-Forget [20] exhibit a much larger performance drop, with ACC decreasing by 9.24 percentage points on ASSISTment2017. These results verify that DRAKT’s dynamic memory filtering and reinforcement learning-based adjustment mechanisms effectively handle challenges posed by data sparsity and distributional differences across datasets.

### 4.4. Ablation Study

To quantitatively validate the contribution of each core component and sub-module in the DRAKT model, a systematic ablation study was conducted based on the model’s two key innovations—Q-learning-based knowledge state adjustment and dynamic memory update with attention-GRU fusion—and four model variants were designed to gradually remove or isolate individual components, disentangling their independent and synergistic effects on performance. All experiments adopted ten-fold cross-validation on the three ASSISTments datasets, with AUC and ACC as evaluation metrics to ensure reliability. The ablation variants are defined as follows:

1. **DRAKT-a**: Neither the knowledge point update module nor the dynamic memory update module is used.
2. **DRAKT-b**: The knowledge point update module is used, while the memory update module is not.
3. **DRAKT-c**: The knowledge point update module is not used, while the dynamic memory update module (with reward adjustment removed) is used.
4. **DRAKT-d**: The knowledge point update module is not used, while the dynamic memory update module is used.

All variants maintain consistent data distribution and experimental settings with the full model to ensure fair comparison, and the results of the ablation study are summarized in Table 3. The results show that the reinforcement learning-based knowledge point update module and the dynamic memory update module play significant roles in improving the performance of the knowledge tracing model. Compared to DRAKT-a, which incorporates neither module, the full DRAKT model achieves a performance improvement of at least 6.83%. The knowledge point update module alone (DRAKT-b) raises accuracy by at least 1.8% over DRAKT-a, while the dynamic memory update module (DRAKT-d) outperforms its static counterpart (DRAKT-c) by at least 0.9%. Ultimately, the integrated DRAKT model further surpasses DRAKT-d, demonstrating that combining both modules enables more effective real-time adaptation to students’ learning processes and yields more accurate predictions of dynamically changing knowledge states.

To verify the contributions of the two core components—the Reinforcement Learning (RL)-based knowledge adjustment module and the dynamic memory update mechanism with reward shaping—we performed ablation experiments using four model variants on the three datasets: DRAKT-a, which removes both the RL module and dynamic memory updates; DRAKT-b, which retains only the RL-based adjustment; DRAKT-c, which employs dynamic memory updates without reward adjustment; and DRAKT-d, which includes dynamic memory updates enhanced with reward adjustment. The results demonstrate that both components are essential to the model’s performance. The full DRAKT model outperforms DRAKT-a by at least 6.83 percentage points in AUC, confirming the synergistic effect of integrating the two modules. Moreover, DRAKT-b achieves an AUC 1.8–2.0 percentage points higher than DRAKT-a, indicating that RL-driven dynamic adjustment improves the accuracy of state tracking. Comparing the memory variants, DRAKT-d surpasses DRAKT-c by 0.9–1.1 percentage points in AUC, verifying that reward-guided memory updates better simulate authentic learning and forgetting processes. Finally, the complete integration in DRAKT yields an additional gain of 1.7–2.0 percentage points in AUC over DRAKT-d, as the reward signal from the RL module further optimizes the memory filtering threshold and update intensity.

In general, the model performs well on the ASSISTment2009 dataset (which has a small data volume and prominent data sparsity), indicating that the memory filtering mechanism equips the DRAKT model with strong capabilities in handling sparse data. For the more complex ASSISTment2012 dataset, some models fail to fully explore the associations between knowledge points; in contrast, DRAKT demonstrates stronger adaptability via its dynamic memory update mechanism, and its performance is significantly superior to that of the comparison models. For the ASSISTment2017 dataset (which has a larger scale and more diverse learning behaviors), most models have limited generalization capabilities. However, DRAKT can effectively handle complex data scenarios by integrating reinforcement learning and dynamic modeling, thus showing excellent universality.

To further verify the impact of the memory filtering threshold θ on model performance, an ablation experiment is designed for the memory filtering threshold parameter θ in the DRAKT model, and conducted on the three datasets. In the experiment, the value of θ is set to 0, 0.1, 0.2, 0.3, 0.4, 0.5, 0.6 in sequence. It should be noted that when θ is too large, a large amount of valid information will be filtered out, thereby preventing the model from fully learning the effective features in the data. The specific experimental results are shown in Table 4.

A sensitivity analysis was conducted on the memory filtering threshold θ—a key parameter of the dynamic memory module—by testing values ranging from 0.1 to 0.6. As shown in Table 4, a threshold of θ = 0.2 achieves the optimal balance between model performance and generalization. When θ is set below 0.2, excessive retention of irrelevant memory entries introduces noise and interferes with prediction, as the model fails to focus sufficiently on knowledge points with higher relevance; this is reflected in the lower AUC of 0.79 on ASSISTment2009 at θ = 0.1. Conversely, when θ exceeds 0.2, overly filtering removes valid knowledge features, thereby reducing the model’s adaptability. This occurs because an excessively high threshold prevents the model from adequately learning the underlying patterns in the data, significantly impairing its generalization ability—for example, at θ = 0.6, AUC drops to 0.77 on ASSISTment2012. Furthermore, as the volume of answer sequences increases, the model requires a stronger filtering intensity to enhance its focus on important knowledge; however, this must be carefully calibrated to avoid the aforementioned pitfalls. In contrast, θ = 0.2 consistently yields the highest AUC across all datasets (ranging from 0.81 to 0.82), confirming that this setting optimally balances the retention of relevant features with the removal of noise, thereby aligning with the principled goal of maintaining both high performance and robust generalization, a conclusion consistent with the results obtained during hyperparameter tuning.

### 4.5. Visualization Analysis

**Figure 4:**
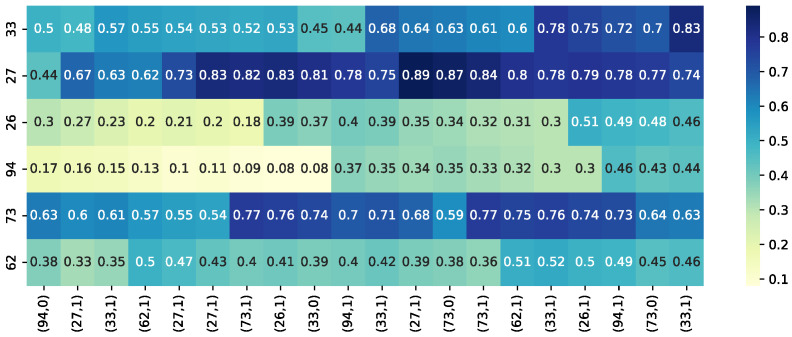
Thermodynamic diagram of student knowledge state evolution process.

To intuitively demonstrate the model’s effect, this paper plots a thermodynamic diagram showing the change in the model’s predicted mastery probability of knowledge points—covering 20 interaction points of 6 knowledge points—for a randomly selected student from the ASSISTment2009 dataset during the answering process. In Figure 4, dark-colored areas represent a high mastery rate, while light-colored areas represent a low mastery rate.

Figure 4 visualizes the predicted knowledge mastery for a randomly selected student from the ASSISTment2009 dataset across six knowledge points and 20 learning interactions, presented as a heatmap (with darker colors indicating higher mastery). The visualization reveals several key behavioral insights captured by the DRAKT model. First, it demonstrates stable knowledge consolidation: for instance, the mastery level of knowledge point 27 rises from 0.44 to over 0.80 after five consecutive interactions and remains high thereafter, indicating that the model effectively recognizes and retains consolidated knowledge. Second, it provides insight into momentous learning gains: the mastery of knowledge point 94 remains low (around 0.15) for the first nine interactions but jumps to 0.37 after the tenth, reflecting a sudden moment of understanding facilitated by the model’s RL-based reward for persistent practice and its dynamic memory, which retains relevant prior context. Finally, it simulates realistic forgetting: the mastery of knowledge point 33 declines from 0.70 to 0.55 when not practiced for eight consecutive steps, a pattern consistent with the Ebbinghaus forgetting curve and confirming the model’s ability to replicate natural knowledge decay over time.

## 5. Conclusions

This paper proposes DRAKT, a knowledge tracing model that integrates reinforcement learning and dynamic memory update. The model adaptively models students’ knowledge states via a reinforcement learning mechanism to achieve accurate evaluation, and effectively captures the state change patterns in the learning process through dynamic memory update and filtering strategies.

Experiments on three public educational datasets show that DRAKT outperforms traditional models in predictability, providing a new “theoretical and application reference” for knowledge tracing and personalized learning recommendation in intelligent education systems. In terms of practical teaching adaptation, DRAKT can well support personalized learning path recommendation and resource reconfiguration.

However, the model currently faces three key challenges: 1. Irregular educational data annotation and privacy protection issues increase the complexity of data processing; 2. Cross-disciplinary knowledge point association phenomena are difficult to visualize, and the accuracy of the existing memory filtering mechanism needs improvement; 3. Closed architectures of some school systems restrict the model’s expansion to large-scale educational data and multi-terminal learning scenarios.

In future work, we will focus on exploring the model’s cross-disciplinary and multi-system compatibility, and further optimize data processing and engineering deployment strategies—to promote intelligent education evaluation toward a more efficient and humanized direction.

## Figures and Tables

**Table 1:** Dataset Overview.

Name	ASSISTment2009	ASSISTment2012	ASSISTment2017
Number of Students	3884	27,485	1708
Number of Exercises	17,737	52,065	3162
Number of Skills	123	265	102
Number of Interaction Records	337,559	2,709,436	942,814

**Table 2:** Prediction Results of Different Models on the Knowledge Tracing Task.

Model	ASSISTment2009		ASSISTment2012		ASSISTment2017	
	AUC	ACC	AUC	ACC	AUC	ACC
BKT [5]	0.62	0.65	0.62	0.65	0.61	0.64
DKT [6]	0.73	0.70	0.72	0.78	0.72	0.69
AKT [12]	0.75	0.72	0.74	0.72	0.74	0.71
DKVMN [8]	0.75	0.73	0.74	0.72	0.71	0.68
LPKT [22]	0.78	0.74	0.78	0.72	0.79	0.71
DKT-Forget [21]	0.74	0.73	0.74	0.72	0.72	0.62
GKT [18]	0.79	0.75	0.78	0.74	0.76	0.74
DRAKT	0.81	0.80	0.81	0.80	0.82	0.76

**Table 3:** Results of Ablation Experiments.

Model	ASSISTment2009		ASSISTment2012		ASSISTment2017	
	AUC	ACC	AUC	ACC	AUC	ACC
DRAKT-a	0.76	0.76	0.76	0.78	0.75	0.76
DRAKT-b	0.78	0.80	0.76	0.78	0.78	0.76
DRAKT-c	0.79	0.79	0.79	0.79	0.78	0.76
DRAKT-d	0.79	0.79	0.79	0.79	0.80	0.76
DRAKT	0.81	0.80	0.81	0.80	0.82	0.76

**Table 4:** The Impact of θ-Value on Model Prediction Results.

	0	0.1	0.2	0.3	0.4	0.5	0.6
ASSISTment2009	0.78	0.80	0.81	0.81	0.81	0.79	0.77
ASSISTment2012	0.79	0.80	0.81	0.80	0.79	0.78	0.77
ASSISTment2017	0.81	0.82	0.82	0.82	0.81	0.81	0.80

## Data Availability

ASSISTMents2009: https://sites.google.com/site/assistmentsdata/home/2009-2010-assistment-data (accessed on 14 May 2025) ASSISTMents2012: https://sites.google.com/site/assistmentsdata/datasets/2012-13-school-data-with-affect (accessed on 14 May 2025) ASSISTMents2017: https://sites.google.com/view/assistmentsdatamining/dataset?authuser=0 (accessed on 14 May 2025).

## Abbreviations

The following abbreviations are used in this manuscript:

DRAKT	Dynamic Reinforcement learning-based Adaptive Knowledge Tracing model
GRU	Gated Recurrent Unit
